# Supplementary material for: Improvement of Barrier Properties of Biodegradable Polybutylene Succinate/Graphene Nanoplatelets Nanocomposites Prepared by Melt Process
Source: Membranes (Basel). 2021 Feb 22;11(2):151. doi: 10.3390/membranes11020151 (PMC7926900; doi:10.3390/membranes11020151)
Supplement: Supplementary file 1 [file membranes-11-00151-s001.pdf]

membranes

Supplementary Material: Improvement of Barrier Properties of Biodegradable Polybutylene Succinate/Graphene Nanoplatelets Nanocomposites Prepared by Melt Process

Raphaël Cosquer, Sébastien Pruvost and Fabrice Gouanvé

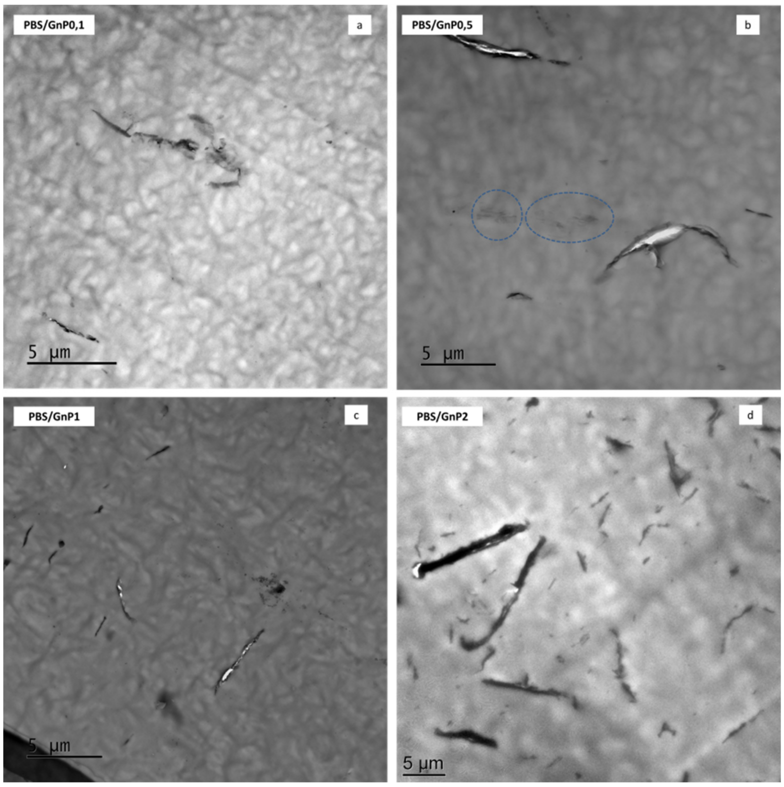

Figure S1. Transmission electron micrographs of nanocomposites films **a**) PBS/GnP0.1; **b**) PBS/GnP0.5; **c**) PBS/GnP1; **d**) PBS/GnP2.

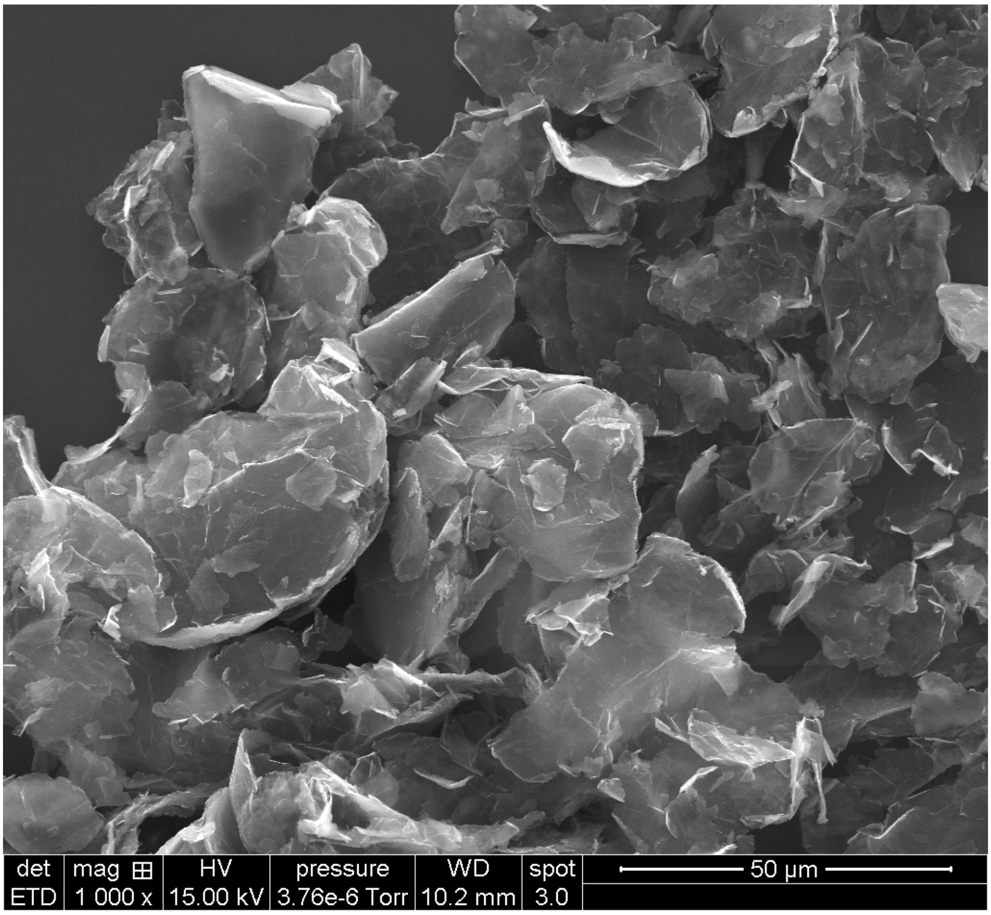

Figure S1 Scanning electron micrographs of GnP particles.

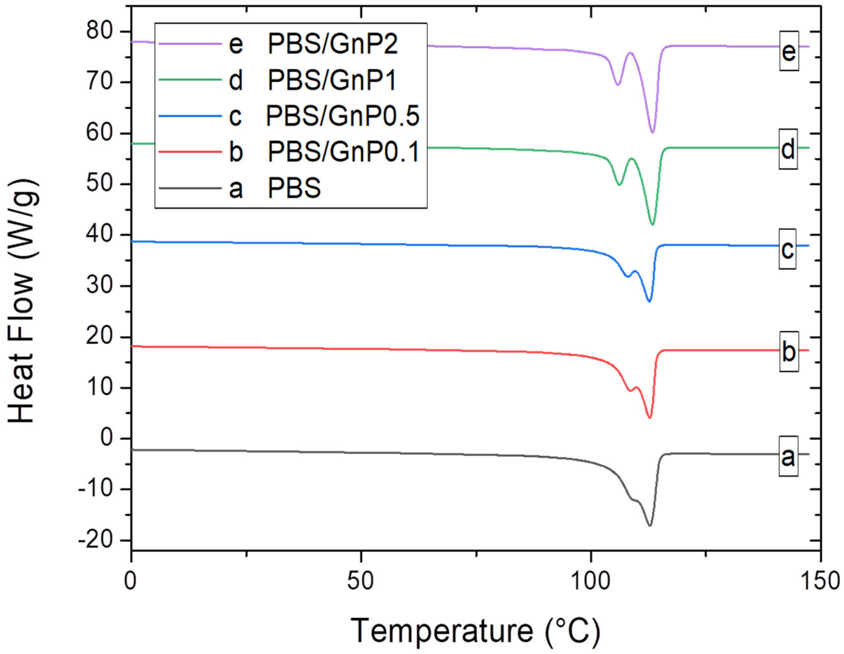

Figure S2 Differential Scanning Calorimetry (DSC) thermograms of second heating scan of neat PBS and corresponding composites.

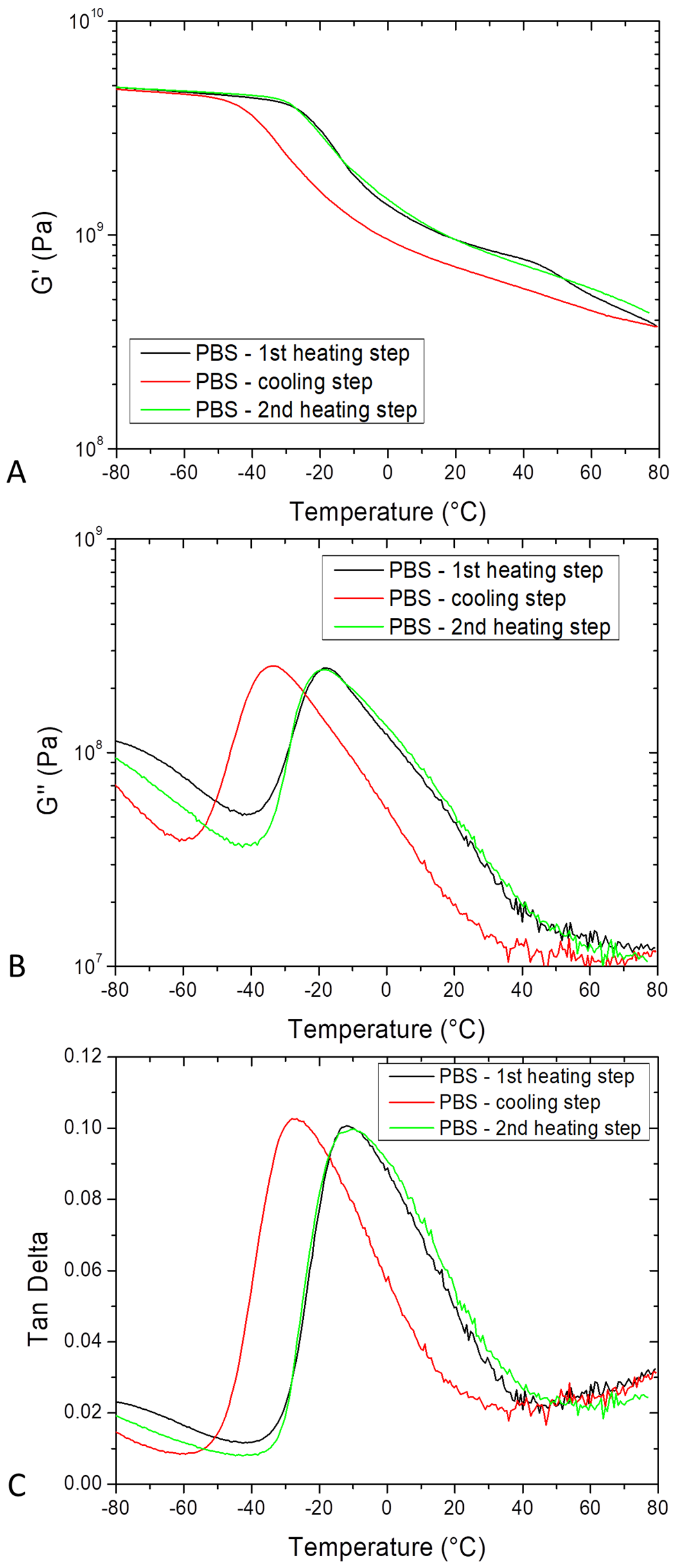

Figure S3 Evolution of (**A**) storage modulus (G′), (**B**) loss modulus (G′′) and (**C**) tan δ as a function of the temperature during first heating step, cooling step and second heating step of neat PBS at 10Hz (range from − 80 °C to 80 °C at 2 °C/min)

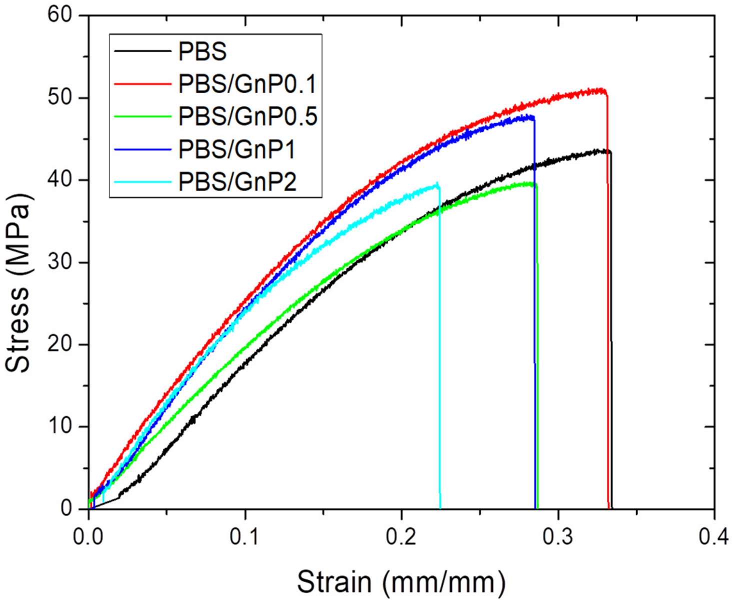

Figure S4 Typical stress - strain curves of neat PBS and corresponding PBS nanocomposites, measured by tensile test.

Table S1. Value of n for the empirical equation (**Error! Reference source not found.**) and the coefficient of determination (R^2) for each activity for neat PBS and corresponding PBS nanocomposites.

	**PBS**		**PBS/GnP0.1**		**PBS/GnP0.5**		**PBS/GnP1**		**PBS/GnP2**	
**a_w**	**n**	**R^2**	**n**	**R^2**	**n**	**R^2**	**n**	**R^2**	**n**	**R^2**
0.1	0.5	0.990	0.5	0.994	0.7	0.997	0.5	0.999	0.5	0.997
0.2	0.5	0.997	0.5	0.997	0.5	0.991	0.6	0.999	0.5	0.997
0.3	0.5	0.994	0.5	0.993	0.6	0.997	0.6	0.998	0.5	0.997
0.4	0.5	0.995	0.5	0.995	0.6	0.997	0.5	0.998	0.5	0.995
0.5	0.5	0.995	0.5	0.994	0.5	0.995	0.5	0.999	0.5	0.997
0.6	0.5	0.997	0.5	0.996	0.5	0.997	0.6	1.000	0.5	0.997
0.7	0.5	0.997	0.5	0.995	0.5	0.997	0.5	0.998	0.5	0.998
0.8	0.7	0.998	0.7	0.997	0.7	0.998	0.8	0.999	0.6	0.996
0.9	0.5	0.996	0.5	0.998	0.5	0.996	0.5	0.997	0.5	0.996
